# 18 Years of Experience in Knee Arthroscopy Under Local Anesthesia

**DOI:** 10.1155/DTE.3.1

**Published:** 1996

**Authors:** W. Birkner, H. R. Henche

**Affiliations:** Orthopädie Am Vogelsang 4 Rheinfelden D-79618 Switzerland

## Abstract

Today, arthroscopies of the knee joint have become a routine procedure. Generally, these operations are performed under general or spinal anesthesia. In Rheinfelden, local anesthesia is our method of choice. We will summarize our experience with nearly 9000 arthroscopies in the last 18 years.

We use 10 mls of ScandicainR as the local anesthetic for the puncture track, followed by an intraarticular injection of 20 mls of CarbostesinR. From 1977 to 1994, arthroscopies were performed on 8720 patients, of which 90% were outpatients, under local anesthesia. When asked about pain sensation, 50% of patients had no pain at all, 34% expressed mild pain and only 3% reported strong pain. 90% of all patients questioned responded that they would undergo the same procedure a second time, if necessary.

There are several advantages of local anesthesia, most important the minimal invasive procedure. Risk factors can be avoided, the pain level described by the patients is acceptable and the costs are lower than with other anesthetizing techniques. As a prerequisite for performing these operations under local anesthesia, the surgeon must be very experienced in the handling and routine of arthroscopic procedures.


*Diagnostic and Therapeutic Endoscopy*, Vol. 3, pp. 1–7
Reprints available directly from the publisher
Photocopying permitted by license only

© 1996 OPA (Overseas Publishers Association)
Amsterdam B.V. Published in The Netherlands
by Harwood Academic Publishers GmbH
Printed in Singapore

### 18 Years of Experience in Knee Arthroscopy Under Local Anesthesia

W. BIRKNER* and H. R. HENCHE

*Orthopädie, Am Vogelsang 4, D-79618 Rheinfelden*

*(Received 20 July 1995; Revised 27 November 1995; In final form 31 January 1996)*

**Today, arthroscopies of the knee joint have become a routine procedure. Generally, these operations are performed under general or spinal anesthesia. In Rheinfelden, local anesthesia is our method of choice. We will summarize our experience with nearly 9000 arthroscopies in the last 18 years.**

**We use 10 mls of ScandicainR as the local anesthetic for the puncture track, followed by an intraarticular injection of 20 mls of CarbostesinR. From 1977 to 1994, arthroscopies were performed on 8720 patients, of which 90% were outpatients, under local anesthesia. When asked about pain sensation, 50% of patients had no pain at all, 34% expressed mild pain and only 3% reported strong pain. 90% of all patients questioned responded that they would undergo the same procedure a second time, if necessary.**

**There are several advantages of local anesthesia, most important the minimal invasive procedure. Risk factors can be avoided, the pain level described by the patients is acceptable and the costs are lower than with other anesthetizing techniques. As a prerequisite for performing these operations under local anesthesia, the surgeon must be very experienced in the handling and routine of arthroscopic procedures.**

*Keywords:* Knee arthroscopy, local anesthesia, arthroscopic operations

### INTRODUCTION

Today, arthroscopies of the knee joint have become a frequently performed routine intervention [2,4,5,8,9, 10,13]. Even larger operative procedures, such as ACL reconstructions, are being performed under arthroscopic control with minimal skin incisions. The minimally invasive approach should not only be applied to the surgical technique, but also be considered when choosing the method of anesthesia. Although most surgeons are accustomed to operating under general or spinal anesthesia [2,4,5,10,16,17,18], local anesthesia complies best with the idea of least traumatization [1,9]. Since establishing the Department of Orthopedics in 1977, we have been using local anesthesia as the method of choice for arthroscopies of the knee joint [7]. We will report our experiences with approximately 9000 knee arthroscopies in the last 18 years.

### METHODS

Under normal circumstances, the patient is examined preoperatively at our outpatient clinic. Consent is obtained after explaining the operation and mentioning

*Corresponding author. Tel.: 07623/94352. Fax: 07623/20459.

the possible risks. On the day of the operation, after shaving and disinfection the knee with Dibromol[R], the intended areas of incision (anterolateral and anteromedial) are infiltrated with 10 mls of Scandicain[R] and 1% epinephrine(1:200 000). The knee is bent into a 90° flexion position and via the anterolateral approach, 20 mls of Carbostesin[R] 0,25% is injected into the joint cavity (Fig. 1). Then time is given for the anesthetic agent to take effect. The patient is asked to move the knee joint in order to improve the intraarticular distribution of Carbostesin[R]. The thigh is placed in a leg holder and draped with sterile sheets in a manner that allows the surgeon and the patient, if desired, to view the monitor. On request, the patient can listen to music ia cordless earphones during the operation.

The arthroscope (30° angle optic) is introduced from an anterolateral approach. After a brief inspection of the joint cavity the probe is introduced from anteromedial in order to palpate the intraarticular structures. The use of a probe is mandatory. Having defined the pathologic mechanism, the arthroscopic operation is performed through the medial access. The anteromedial approach is rarely used. Usually, we insufflate $CO_2$, as a gaseous medium, into the knee joint. It is important to use a pressure regulator to regulate the gas pressure and a controller to limit gas quantity [9]. The arthroscopic operation is performed immediately under the same local anesthesia. Generally, the visual field under these circumstances is excellent, so operations on the menisci, cartilage and synovia can be accomplished accurately.

### RESULTS

From 1977 to 1994, 8720 arthroscopies of the knee joint were performed (Fig. 2) Since 1977 the number of arthroscopies has been increasing steadily, reach-

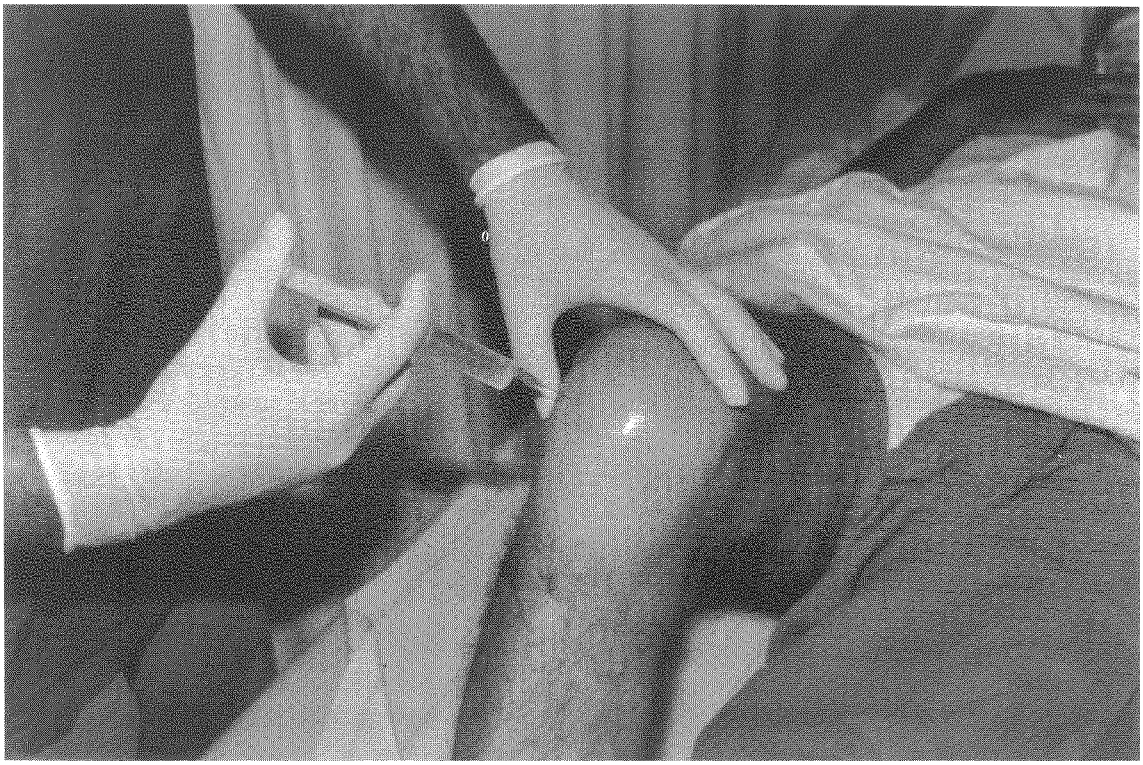

FIGURE 1 Injection of Carbostesin[R] into the joint cavity.

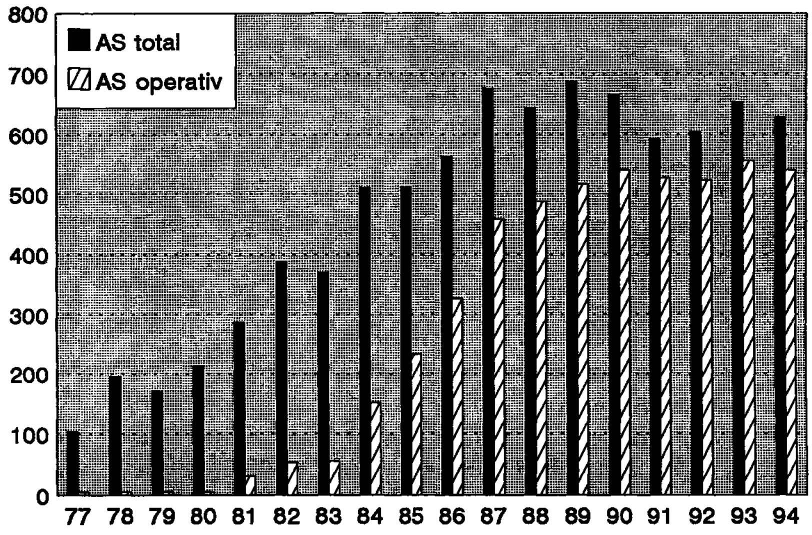

FIGURE 2 Number of arthroscopies 1977–1994.

ing a peak capacity of approximately 700 arthroscopies per year in the late 1980s. A lack of operative capacity made a further increase impossible. Furthermore, critical indication for arthroscopy was strongly emphasized.

During the time period reviewed ninety percent of all arthroscopies were completed using local anesthesia. This high percentage of arthroscopies under local anesthesia has remained constant over the years. However since 1994, arthroscopies under general anesthesia have increased due to the increasing number of normal or less extensive arthroscopies being performed in outpatient clinics and practices. Arthroscopies performed in our hospital are now generally more complex procedures, such as meniscus repair, reconstruction of the anterior crucial ligament (ACL), and synovectomy.

Ten years ago, only 13% of all arthroscopies were combined with an immediate operative therapy. To a large extent this was due to the the lack of experience of the performing orthopaedic surgeon, who did not have a reliable diagnosis beforehand. Also, the technology of surgical techniques and instruments was less developed. Often, the exact diagnosis was only made during the arthroscopy. If the arthroscopic findings indicated that an operation was necessary, the patient was hospitalized after a few days and an arthrotomy was performed under general or spinal anesthesia. Today, only about 15% of all arthroscopies are solely for diagnostic purposes. In the remaining 85% the arthroscopic surgeries are performed immediately under local anesthesia. Additionally, we analyzed all arthroscopic diagnoses from 1989 until 1990. The most common findings were lesions of the medial meniscus (67%), 45% of these were flap-like tears, 35% were degenerative menisci, and 20% were bucket-handle tears. 22% of the cases required an operation of the lateral meniscus.

The duration of the arthroscopy in 60% of all cases was under 30 minutes, only 3% exceeded 60 minutes. The latter primarily due to technically difficult lesions of the posterior horn of the meniscus.

The anticipated pain level during the operation is a major factor when considering an arthroscopy under local anesthesia. In 1990, we questioned 163 patients. 50% reported absolutely no pain during arthroscopy, 34% described light or moderate pain, 13% experienced strong pain, and 3% complained of extreme pain (Fig. 3). Patients with pain were then asked to specify which parts of the procedure caused the most pain. 20% claimed that the injection of the local anesthetic was painful, 34% complained of pain during

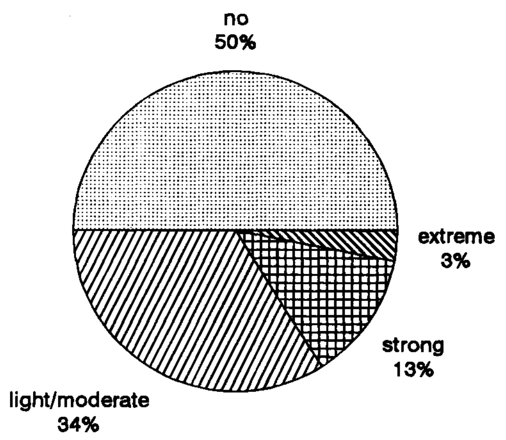

FIGURE 3 Intensity of pain during arthroscopy in local anesthesia.

forced valgus position in the leg holder, and 27% described the intraarticular manipulation of the injured meniscus as painful. The remaining 20% of the resonses were variable, e.g. inserting the arthroscope or back pain due to an uncomfortable position during arthroscopy.

When asked if they would prefer local anesthesia in case of a future arthroscopy, 90% of all patients answered "yes", 7% said "no", and 3% were undecided.

### DISCUSSION

Knee arthroscopies performed under local anesthesia are currently not standard practice. In a recent multicenter study analyzing over 50 000 analysed arthroscopies Tilling [17] found that only approximately 5% of all arthroscopies were performed under local anesthesia. Some authors always use general or regional anesthesia and only employ local anesthesia in exceptional cases [2,4,5,10,13,16,18]. Nevertheless, we believe that local anesthesia is a reasonable method for routine knee arthroscopies today [1,9,14]. More than 20 years ago, Henche published one of the first articles on knee arthroscopies in the German literature [6]. He claimed that general anesthesia was necessary in most cases based on the limited experience with arthroscopies at the time and subsequently the frequent necessity for arthrotomy.

When arthroscopy was introduced at the Orthopedic department in Rheinfelden 18 years ago, the main reason for using local anesthesia was the ability to carry out arthroscopies on our outpatients in an uncomplicated manner (Table I). Due to an insufficient inpatient capacity at the hospital, inpatient arthroscopies using general or regional anesthesia were difficult to accomplish. Also, the use of general anesthesia required the hospitalization of patients one day prior to the operation, as well as a one to two day postoperative stay. Today, these requirements have changed. Given the use of modern anesthetic drugs and an efficient organization, outpatient operations are easily possible [4]. Nevertheless, there is a risk of complications when using general anesthesia. Comparing mortality rates of arthroscopies under general versus local anesthesia, Kieser states a 5:1 ratio [11]. Patients often suffer from nausea following endotracheal anesthesia, and younger patients commonly complain about postspinal head aches after spinal anesthesia [12].

The amount of local anesthetics used in our clinic is far below the maximal doses. Side effects, such as petit-mal attacks, only occur in much higher dosage ranges [12,14]. One of the great advantages of local anesthesia is the ability of the patient to follow the operation on the monitor. This allows the patient to participate in the decision-making when unexpected pathologic findings suggest further arthroscopic intervention. Another complication that can occur under general anesthesia is ligament injuries, such as the medial collateral ligament [3]. This type of injury cannot occur under local anesthesia, since the patient gen-

TABLE I Advantages of Local Anesthesia

| | |
|---|---|
| 1. | Easily performed on outpatients |
| 2. | Cost effective |
| 3. | Anesthetist not required |
| 4. | Minimally invasive method |
| 5. | No anesthesia associated risks |
| 6. | No postspinal headache<br>no postoperative nausea |
| 7. | Intraoperative communication with patient possible<br>(also possible under spinal anesthesia) |
| 8. | No mistakes of operation site possible |
| 9. | No iatrogenic injury to collateral ligaments |

erally does not tolerate extreme valgus strain during arthroscopy. The risk of mistakenly operating on the wrong side is also minimized by a conscious patient.

The application of $CO_2$ as a gaseous, intraarticular medium enables the surgeon to inspect all structures under excellent conditions (FIG 4), without the magnifying "aquarium effect" [9] that occurs when using a liquid medium. So far, no complications have been documented when using $CO_2$ as a gaseous medium. However, complications were seen in earlier attempts with insoluable nitogen gas [11]. Even arthroscopic operations can be performed easily in a $CO_2$ medium. However, we have experienced loss of the visual field during operations exceeding approximately 30 minutes, primarily due to intraarticular bleeding. Edema caused by leaking intraarticular liquid does not occur and escaping $CO_2$ gas is absorbed rapidly. Additionally, we have used $CO_2$ successfully for arthroscopies of other joints, such as the shoulder, elbow, and ankle. A short arthroscopy with $CO_2$ gas makes a precise diagnosis possible and the procedure can be followed by immediate arthrotomy.

Cost has become an increasingly important factor in health care. There is no doubt that arthroscopies performed under local anesthesia are much more cost effective, especially if all extra costs are taken into consideration. In Rheinfelden, for example, no anesthesist is required, because the surgeon injects the local anes-

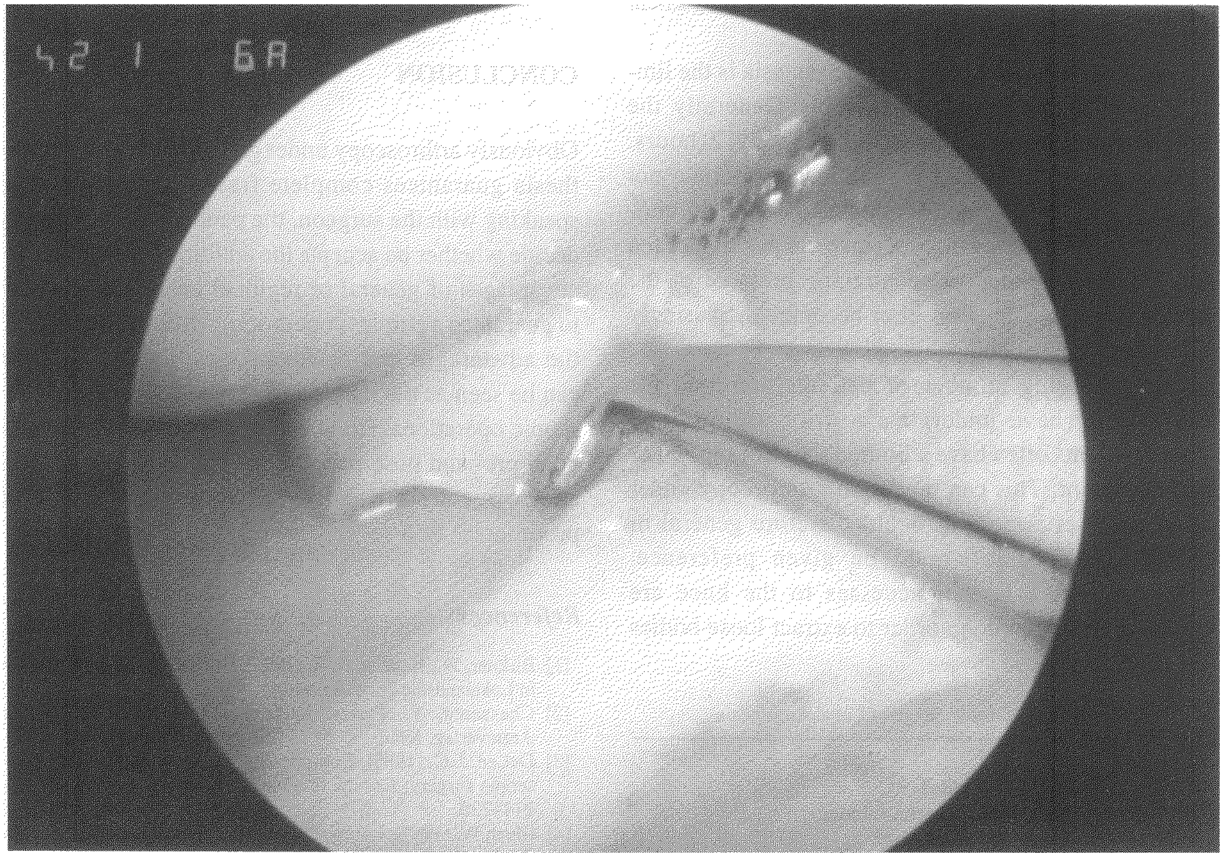

FIGURE 4 Flaplike tear of the medial meniscus under $CO_2$.

thetic prior to the operation. Recent cost analyses in the United States have reached similar conclusions [15].

Naturally, it is important to consider the disadvantages of operating under local anesthesia (Table II). Diagnostic and minor arthroscopic interventions such as meniscus and cartilage surgery, extraction of loose bodies, biopsy of the synovia, or plica resection can be performed easily under local anesthesia. On the other hand, meniscal repair or ACL reconstructions are difficult or impossible under these conditions. This merely emphasizes the importance of a complete medical history and preoperative physical examination prior to the procedure. For example, if a young patient has clinical signs of a bucket-handle tear, we would opt for an arthroscopy under general or regional anesthesia, since a repair of the torn meniscus would remain possible. The same strategy would be applied in the case of ligament injuries to the knee joint. A possible instability must be assessed before arthroscopy, in case a ligament reconstruction should be required.

Another disadvantage of local anesthesia is the limited time available for arthroscopy. Generally the anesthetic effect lasts for approximately 2 hours. However, the duration of the arthroscopy should not exceed much more than 30 minutes. We see 60 minutes as an absolute maximum, since the position of the leg in the leg holder and continuing pressure of the knee joint in a valgus position becomes increasingly painful for the patient.

An increasing number of patients admitted for arthroscopy have undergone a prior arthroscopy. These patients often have a limited range of motion of the knee joint. This can render an arthroscopy under local anesthesia more difficult, therefore general or regional anesthesia should be given preference. Likewise, when further accesses to the knee are required, for example in order to extract loose bodies in the dorsal recess, general or regional anesthesia should still be seen as the method of choice.

TABLE II Disadvantage of Local Anesthesia

| | |
|---|---|
| 1. | Possible pain |
| 2. | Operation cannot be extended (e.g. Meniscal repair, ACL reconstruction) |
| 3. | No muscle relaxation (difficult for operations of the posterior horn of the meniscus) |
| 4. | Not suitable for inexperienced surgeons |

Generally, most surgeons prefer to operate under general or regional anesthesia, because pain is not a consideration and mor comfortable joint manipulation is possible. When operating at difficult locations such as the posterior horn of the meniscus, extreme valgus strain does not trigger pain or muscular tension. Also, it is always possible to obtain an optimal field of vision.. On the other hand, we have experienced that with sufficient local anesthetic and adequate psychological assistance even more difficult arthroscopic operations can be accomplished.

When operating under general or regional anesthesia, it is possible to apply a pneumatic tourniquet. However, most arthroscopic operations do not cause any significant bleeding, so the use of a tourniquet is unnecessary.

### CONCLUSION

Obviously arthroscopy under general or regional anesthesia guarantees complete freedom of pain. After speaking with the surgeon, the patient must ultimately decide whether he accepts the additional risks and disadvantages of general or regional anesthesia in order to guarantee a painless operation. After considering all the advantages and disadvantages, local anesthesia can be seen as the method of choice for minor arthroscopic operations. However, this is only feasible with good pre- and postoperative patient care and an operating surgeon sufficiently experienced in arthroscopic procedures.

### *References*

[1] Birkner, W., Henche, H. R. (1991). Die Kniegelenkarthroskopie in Lokalanästhesie, *Arthroskopie*, **4**, 122–126.
[2] Chassaing, V., Parier, J. Arthroskopie des Kniegelenks. Ärzteverlag, Köln.
[3] DeLee, J. C. (1985). Complications of arthroscopy and arthroscopic surgery: Results of a National Survey, *Arthroscopy*, **1**, 214–220.
[4] Glinz, W. (1987). Diagnostische Arthroskopie und arthroskopische Operationen am Kniegelenk, 2. Auflage. Huber, Bern.
[5] Hempfling, H. (1989). Einführung in die Arthroskopie. Gustav-Fischer Verlag, Stuttgart-New York.

[6] Henche, H. R. (1974). Indikation, Technik und Resultate der Arthroskopie nach Traumatisierung des Kniegelenkes, *Orthopäde,* **3**, 178–183.
[7] Henche, H. R. (1981). Die Arthroskopie. Beitr. Orthop. u. Traumatol, **28**(7), 363–372.
[8] Henche, H. R. (1990). Die arthroskopische Meniskusresektion. *Orthopäde,* **19**, 77–81.
[9] Henche, H. R., Holder J. (1988). Die Arthroskopie des Kniegelenkes, 2. Auflage. Springer, Berlin-Heidelberg- New York.
[10] Johnson, L. (1981). Diagnostic and surgical arthroscopy. The knee and other joints. The C.V. Mosby Company, St. Louis-Toronto-London.
[11] Kieser, Ch. (1989). Übersicht über die schwerwiegenden Komplikationen der Arthroskopie. In: Fortschritte in der Arthroskopie, Bd 5. Enke, Stuttgart, S 1–9.
[12] Larson, R. (1985). Anästhesie. Urban & Schwarzenberg, München- Wien- Baltimore.
[13] Löhnert, J., Raunest, J. (1985). Arthroskopische Chirurgie dees Kniegelenkes. Regensberg& Biermann, Münster.
[14] Mohr, M., Hey, W., Henche, H. R. (1989). Komplikationen bei Lokalanästhesie und Gasinsufflation und ihre Beherrschung. In: Fortschritte in der Arthroskopie, Bd. 5, Enke, Stuttgart, S. 48–53.
[15] Shapiro, M. S., Safran, M. R., Crockett, H., Finerman, G. (1995). Local anesthesia for knee Arthroscopy. Efficacy and Cost Benefits, *Am J Sports Med.,* **23**(1), 50–53.
[16] Strobel, M. *et al.* (1989). Arthroskopische Untersuchung des Kniegelenkes. Ärzteverlag, Köln.
[17] Tilling, Th. (1994). Analyse von 50.000 Kniegelenkarthroskopien—Eine multizentrische, prospektive Datenerhebung der SFA. Stiftung zur Förderung der Arthroskopie, Tuttlingen.
[18] Watanabe, M., Takeda, S., Ikeuchi, H. (1979). Atlas of Arthroscopy. 3. Aufl. Springer, Berlin-Heidelberg-New York.

